# Health system barriers to HPV and MMR vaccination in recent migrants/refugees in Greece: Pania Karnaki

**DOI:** 10.1093/eurpub/ckac129.187

**Published:** 2022-10-25

**Authors:** P Karnaki, A Dalma, N Papaevgeniou, K Katsas, A Linos

**Affiliations:** Prolepsis, Institute of Preventive Medicine Environmental and Occupational Health, Marousi, Greece

## Abstract

**Background:**

Migrants have lower vaccination rates compared to the general population and report multiple barriers in accessing related services. We explored practices and perceptions regarding MMR & HPV vaccinations in migrant children & adolescents from third countries to inform the development of tailored interventions to increase vaccination coverage. Third country nationals for the RIVER-EU project are migrants arriving to the EU from countries beyond Europe (the Middle East, Asia & Africa) escaping civil conflict, war, and poverty.

**Methods:**

A qualitative study was conducted in the wider Athens area as part of the RIVER-EU project. Four Focus Groups and 23 semi-structured interviews were conducted with health care professionals, children and parents with a migrant background. Data were analysed using thematic content analysis. Findings: Identified barriers relate to the lack of standard operational procedures at system level that would define a schedule of vaccinations for migrants. Migrant vaccinations are subject to availability of vaccines (MMR as opposed to HPV) and potential threat of outbreaks (MMR vs HPV). There is no consistent, unified recording system of vaccinations while at system level there is a lack of trained cultural mediators. Targeted health promotion campaigns are rare while the few related activities that do exist are not systematically evaluated.

**Conclusions:**

MMR vaccination is more frequent compared to HPV which is not prioritised by the target group or health professionals. Nevertheless, the target group is open to learning more about HPV while the important role of mothers concerning vaccinations emerged as crucial. Health professionals focus more on MMR due to the availability of the vaccine and the threat of outbreaks. The vaccination system has flaws and inconsistencies with a lack of vaccination related data. There is urgent need for culturally appropriate vaccination and appropriately evaluated vaccination campaigns.

